# Memantine and graded motor imagery for complex regional pain syndrome (MEMOIR): study protocol and statistical analysis plan for a decentralised, 2 × 2 factorial randomised trial

**DOI:** 10.1186/s13063-025-09383-8

**Published:** 2025-12-29

**Authors:** Michael C. Ferraro, Yannick L. Gilanyi, Eric J. Visser, Andrew J. McLachlan, G. Lorimer Moseley, Benedict M. Wand, Neil E. O’Connell, Hopin Lee, Martin Lotze, Jody Church, Stephen Goodall, Robert D. Herbert, Sarah E. Lamb, Sylvia M. Gustin, Aidan G. Cashin, James H. McAuley

**Affiliations:** 1 School of Health Sciences, Faculty of Medicine and Health, University of New South Wales, Sydney, Australia; 2 Centre for Pain IMPACT, Neuroscience Research Australia, Randwick, NSW Australia; 3 Faculty of Medicine, Nursing and Midwifery and Health Sciences, The University of Notre Dame Australia, Fremantle, Australia; 4 Sydney Pharmacy School, Faculty of Medicine and Health, The University of Sydney, Sydney, NSW Australia; 5 IIMPACT in Health, University of South Australia, Kaurna Country, Adelaide, Australia; 6 Department of Health Sciences, Centre for Health and Wellbeing Across the Lifecourse, Brunel University of London, Uxbridge, UK; 7 Exeter Medical School, University of Exeter, Exeter, UK; 8 IQVIA, London, UK; 9 Functional Imaging Unit, Center for Diagnostic Radiology, University of Greifswald, Greifswald, Germany; 10 Centre for Health Economics Research and Evaluation, Faculty of Health, University of Technology Sydney, Broadway, NSW Australia; 11 Geebung Statistical Consulting, Blackheath, NSW Australia; 12 NeuroRecovery Research Hub, School of Psychology, University of New South Wales, Sydney, NSW Australia

**Keywords:** Complex regional pain syndrome, Memantine, Graded motor imagery, Factorial, Chronic pain

## Abstract

**Background:**

Complex regional pain syndrome (CRPS) is a rare chronic pain condition characterised by severe pain, sensory, motor, autonomic, and trophic abnormalities. Effective treatment options are limited, and international guidelines rely on low-quality evidence and consensus. Two interventions—memantine, an N-methyl-D-aspartate receptor antagonist, and graded motor imagery, a rehabilitation approach targeting sensorimotor processing—have shown promise in pilot studies but lack definitive evaluation in large-scale trials. MEMOIR aims to evaluate the benefits and harms of memantine and graded motor imagery for CRPS.

**Methods:**

MEMOIR is a fully decentralised, 2 × 2 factorial, randomised trial comparing memantine with placebo and graded motor imagery with no graded motor imagery in adults with CRPS. A total of 204 participants with CRPS of 6 months to 5 years duration will be randomised to one of four groups: (i) memantine and graded motor imagery, (ii) memantine only, (iii) placebo and graded motor imagery, or (iv) placebo only. Memantine will be administered at 40 mg/day (or maximum tolerated dose); graded motor imagery will comprise seven 1-h sessions delivered via telehealth. The treatment period is 16 weeks. The dual primary outcomes are pain intensity (11-point numeric rating scale) and PROMIS pain interference assessed at 16 weeks. Secondary outcomes include physical function, fatigue, cognitive function, depressive symptoms, self-efficacy, health-related quality of life, CRPS severity, healthcare use, and adverse events. The follow-up period is 52 weeks. The estimands of interest are the mean effect of memantine compared to placebo and the mean effect of graded motor imagery compared to no graded motor imagery on all outcomes. All analyses will follow the intention-to-treat principle.

**Discussion:**

MEMOIR will be the largest investigator-initiated CRPS trial to date. The 2 × 2 factorial design and decentralised delivery aim to maximise efficiency, accessibility, and equity. If effective, memantine and graded motor imagery represent scalable, low-cost treatments that can be given in clinics or at home, with the potential to transform CRPS management.

**Trial registration:**

Australian New Zealand Clinical Trials Registry (ACTRN12621000175875). Registered on 12 February 2021.

**Supplementary Information:**

The online version contains supplementary material available at 10.1186/s13063-025-09383-8.

## Administrative information

**Table:** 

Title {1}	Memantine and graded motor imagery for complex regional pain syndrome (MEMOIR): study protocol and statistical analysis plan for a decentralised, 2 × 2 factorial randomised trial
Trial registration {2a and 2b}	Australian New Zealand Clinical Trials Registry: ACTRN12621000175875
Protocol version {3}	Version 8.0; 28 August 2025
Funding {4}	National Health and Medical Research Council of Australia (NHMRC) grant number APP1163149
Author details {5a}	1. School of Health Sciences, Faculty of Medicine and Health, University of New South Wales, Sydney, Australia 2. Centre for Pain IMPACT, Neuroscience Research Australia, Randwick, New South Wales, Australia 3. Faculty of Medicine, Nursing and Midwifery and Health Sciences, The University of Notre Dame Australia, Fremantle, Australia 4. Sydney Pharmacy School, Faculty of Medicine and Health, The University of Sydney, Sydney, New South Wales, Australia 5. IIMPACT in Health, University of South Australia, Kaurna Country, Adelaide, Australia 6. Department of Health Sciences, Centre for Health and Wellbeing Across the Lifecourse, Brunel University of London, Uxbridge, UK 7. Exeter Medical School, University of Exeter, Exeter, UK 8. IQVIA, London, UK 9. Functional Imaging Unit, Center for Diagnostic Radiology, University of Greifswald, Greifswald, Germany 10. Centre for Health Economics Research and Evaluation, Faculty of Health, University of Technology Sydney, Broadway, New South Wales, Australia 11. Geebung Statistical Consulting, Blackheath, New South Wales, Australia 12. NeuroRecovery Research Hub, School of Psychology, University of New South Wales, Sydney, New South Wales, Australia
Name and contact information for the trial sponsor {5b}	Neuroscience Research Australia 139 Barker Street Randwick, NSW, 2031 Australia Contact: Deborah McKay Research Governance and Compliance Manager E: d.mckay@neura.edu.au T: +61 2 9399 1676
Role of sponsor {5c}	The sponsor and funder have no role in study design; collection, management, analysis, and interpretation of data; writing the report; and the decision to submit the report for publication; and will have no authority over any of these activities

## Introduction

### Background and rationale {6a}

Complex regional pain syndrome (CRPS) is a rare pain disorder that usually occurs in a single limb following trauma, such as fracture or surgery [1]. CRPS is characterised by severe pain and sensory, vasomotor, sudomotor, motor and trophic signs and symptoms [1, 2]. The pathophysiological mechanisms of CRPS are incompletely understood, but likely involve aberrant inflammatory and immune responses, vasomotor dysfunction, nervous system changes, genetic variations, and psychological processes [3]. CRPS is classified as a chronic primary pain disorder in the International Classification of Diseases Volume 11 (ICD-11) [2, 4].

CRPS incidence is estimated at between 6 and 26 per 100,000 person-years, meeting US Food and Drug Administration and European Medicines Agency rare disease definitions [5–8]. The disorder is most common in people aged between 50 and 80 years, and affects four females for every male [3]. Data from prospective studies are limited but suggest that while CRPS typically improves rapidly in the first 6 months following symptom onset [9, 10], ongoing pain and motor dysfunction at 12 months and beyond are common [11]. People with persistent CRPS report poor quality of life [12], increased depressive symptoms [13], and reduced work status [11].

Effective treatment options for CRPS are limited. International clinical guidelines recommend multidisciplinary care that includes pharmacological, interventional, psychological, and rehabilitation therapies [14]. However, recommendations are informed by low-quality evidence and consensus. A 2023 Cochrane overview of systematic reviews identified no interventions for CRPS whose efficacy or effectiveness are supported by high- or moderate-certainty evidence [15]. This overview highlighted a critical need for adequately powered, high-quality randomised controlled trials of pharmacological and non-pharmacological interventions, selected based on rigorous pilot research or major clinical uncertainty [15]. It also identified preliminary evidence of effectiveness for two interventions: memantine and graded motor imagery [16–18].

Memantine is a non-competitive N-methyl D-aspartate receptor antagonist (NMDA) approved for treatment of moderate-to-severe Alzheimer’s disease. It is proposed to reduce pain intensity by blocking sustained glutamatergic NMDA receptor activity in central nervous system nociceptive pathways [19–21]. A pilot trial of 20 patients with CRPS showed that, compared with placebo, a 7-week course of 40 mg/day of oral memantine provided clinically important reductions in pain intensity with no between-group differences in adverse events [16]. However, that study was not powered to robustly evaluate efficacy and safety.

Graded motor imagery is an established rehabilitation intervention for CRPS. It aims to reduce pain and improve function through a graded progression of movement imagery, mirror therapy, and functional tasks that target cortical processing of the affected limb [22]. A 2022 Cochrane review found that, compared with treatment as usual, a 6-week graded motor imagery program provided clinically important reductions in pain intensity (mean difference [on a 0–100 scale] −21.0 [95% confidence interval −31.2 to −10.9], 2 studies; 49 participants) [23]. However, previous studies were small, at high risk of bias, and did not assess long-term effects. Despite endorsement in international clinical guidelines [24–27] and widespread use, uncertainty regarding the effectiveness of graded motor imagery remains.

MEMOIR (MEmantine and graded MOtor Imagery for complex Regional pain syndrome) aims to evaluate the benefits and harms of memantine and graded motor imagery for CRPS.

### Objectives {7}

The primary objectives of the MEMOIR trial are to estimate the effects of (i) memantine compared to placebo and (ii) graded motor imagery compared to no graded motor imagery, on pain intensity and pain interference at 16 weeks. Secondary objectives are to estimate the sustained effects of memantine and graded motor imagery on pain intensity and pain interference at 26 and 52 weeks, and immediate and sustained effects of the following outcomes at 16, 26, and 52 weeks: physical function, fatigue, self-efficacy to manage symptoms, cognitive function, depressive symptoms, health-related quality of life, pain self-efficacy, patient global impression of change, CRPS severity score, healthcare use, and adverse events.

### Trial design {8}

MEMOIR is a fully decentralized, 2 × 2 full-factorial, randomized trial with four parallel groups allocated at a 1:1:1:1 ratio to (i) memantine and graded motor imagery; (ii) memantine only; (iii) placebo and graded motor imagery; and (iv) placebo only. All outcomes for each main comparison (memantine versus placebo; graded motor imagery versus no graded motor imagery) are tested for superiority. A 2 × 2 factorial design was chosen because there is little reason to expect an interaction between interventions (that is, there is little reason to expect the effects of either intervention, expressed as an absolute difference, depend on the presence or absence of the other intervention). In the absence of an interaction between interventions, a factorial design provides an efficient way to evaluate the effects of two interventions. This protocol and statistical analysis plan is reported in accordance with the Standard Protocol Items: Recommendations for Interventional Trials (SPIRIT) statement and the extension for factorial randomized trials [28–30].

## Methods: participants, interventions, and outcomes

### Study setting {9}

MEMOIR is a decentralised trial (i.e. a trial that uses procedures conducted outside the traditional clinical trial site) [31, 32], conducted in the community, Australia-wide. Participants will undergo all recruitment, screening, and treatment activities at their home setting.

### Eligibility criteria {10}

#### Inclusion criteria

- Diagnosis of unilateral CRPS according to the Budapest research criteria, of 6 months to 5 years duration
- At least moderate pain and disability as measured using the Short Form Health Survey [33] items 7 and 8
- Aged 18 years and older
- English language proficiency
- Access to internet
- Willingness to provide informed consent and to comply with the study requirements

#### Exclusion criteria

- Women who are pregnant or lactating
- Participants who are female, of child-bearing potential and not using reliable contraceptive method(s)
- Males and females planning conception
- Known allergies to NMDA receptor antagonists
- Using >60 mg/day morphine equivalents of opioid analgesics
- Using methadone
- Using >450 mg/day of pregabalin or >1200 mg/day gabapentin
- Using monoamine oxidase inhibitors
- Using >50 mg/day of tricyclic antidepressants
- Used ketamine in the preceding 4 weeks
- Received lidocaine injections or infusions in the preceding 4 weeks
- Commenced bisphosphonate treatment in the preceding 4 months
- Using anti-arrhythmic cardiac medications
- Using anti-psychotic medications
- Using cisapride, erythromycin, or quinolones
- Using anti-tuberculous or anti-fungal agents
- Using antiretrovirals
- Moderate to severe renal impairment (defined as estimated glomerular filtration rate below 70 mL/min/1.73 m^2^)
- Prolonged QTc syndrome, ventricular fibrillation, ventricular tachycardia, heart block, or Torsades de Pointes
- Acute coronary syndrome in the preceding 3 months
- NYHA classes III–IV heart failure
- Uncontrolled hypertension
- Pacemaker or implantable defibrillator
- History of neurological conditions (e.g. seizure disorders, Alzheimer’s disease, paralysis, stroke)
- History of schizophrenia, psychosis, bipolar disorder, or clinically significant delusions, hallucinations, or delirium
- Significant history of illicit substance abuse or drug overuse
- Implanted spinal cord or nerve stimulators
- Other pain that may interfere with assessment of CRPS and/or use of graded motor imagery, according to the study physician
- Currently using graded motor imagery
- Scheduled for major surgery during the treatment or follow-up period

Participants must meet all inclusion criteria to allow randomisation to all treatment factors. Participant screening is conducted in 3 stages: (i) online using a REDCap screening form [34, 35]; (ii) via telephone with a trained research assistant; and (iii) via telehealth with a specialist pain physician.

### Who will take informed consent? {26a}

Potential participants will receive a written participant information statement that outlines the study objectives, procedures, and potential risks and benefits. They will be instructed to take at least 24 h to read the information statement and discuss their participation in the trial with a relative or a friend. A research assistant trained in ICH Good Clinical Practice [36] and the consent procedures will discuss the trial in light of the information provided in the statement and answer any questions about the trial the potential participant might have. Participants willing to consent will be provided with a REDCap eConsent form. Upon completion of the REDCap eConsent form, a static PDF copy of participant consent responses will be stored in the REDCap project’s file repository and emailed to the participant. The consent-specific PDF will have the values of the eConsent framework options inserted in each page of the PDF. These values (i.e. name, date of birth) are added to the PDF as documentation of the identity of the person who is consenting. Participants enrolled in the study will also have the opportunity to provide consent to Services Australia for the release of Medicare Benefits Schedule (MBS) and/or Pharmaceutical Benefits Scheme (PBS) data to allow for healthcare use and cost-effectiveness evaluations.

### Additional consent provisions for collection and use of participant data and biological specimens {26b}

Participants may be invited to participate in an ancillary qualitative study investigating the acceptability of the trial interventions. Willing participants will undergo a separate informed consent process for this study.

## Interventions

### Explanation for the choice of comparators {6b}

An inert placebo will be used as a comparator for memantine to separate the effect of this medicine from non-specific effects of being allocated and taking an oral medicine. Graded motor imagery will be evaluated against no graded motor imagery, as it was not considered possible to implement a sham that is both convincing and inert.

### Intervention description {11a}

#### Memantine

Oral memantine will be administered at 40 mg/day, or maximum tolerated dose, for 16 weeks, including titration and taper periods. The proposed dose is greater than that approved for moderate-to-severe Alzheimer’s disease (20 mg/day) and is informed by the protocol used by Gustin et al. [16]. By titrating to the highest tolerated dose up to 40 mg/day, the potential analgesic effects can be maximised while minimising the risk of adverse effects. The memantine dosing schedule comprises a 4-week titration period (5 mg/day for 4 days, 10 mg/day for 4 days, 15 mg/day for 4 days, 20 mg/day for 4 days, 25 mg/day for 4 days, 30 mg/day for 4 days, 35 mg/day for 4 days), an 8-week maintenance period (40 mg/day or maximum tolerated dose), and a 4-week taper period (5 mg reduction every 5th day). Memantine tablets are 10 mg, dosed morning and evening.

#### Placebo memantine

Placebo will be dosed according to the same schedule as memantine, including titration and taper periods, and dose individualisation. Placebo memantine tablets will differ from active memantine tablets only in the absence of the active ingredient and will be indistinguishable by sight, touch, or taste. The matched placebo is PROSOLV EASYtab SP (JRS Pharma, Weissenborn, Germany). It consists of 95–98% microcrystalline cellulose, 1.5–2.5% colloidal silicon dioxide, 0.5–2% sodium starch glycolate, and 0.3–1.0% sodium stearyl fumarate.

Trial medicines will be manufactured, packaged, and labelled to comply with Good Manufacturing Practice. The trial medicine will be dispensed by an independent trial pharmacist direct to participants’ homes. Each trial medicine kit will include a pill cutter to halve tablets during titration and taper periods, a medication schedule, and medication instructions.

#### Graded motor imagery

Graded motor imagery is a progressive rehabilitation program thought to sequentially activate brain regions associated with motor planning and execution. The components of the graded motor imagery program include (i) left/right limb judgments (implicit motor imagery), (ii) imagined limb movements (explicit motor imagery), (iii) mirror therapy, and (iv) functional re-engagement and loading of the limb. See [22] for additional detail. The intervention is supported by education about CRPS and patient-led goal-setting.

Participants will individually receive 7, 1-h sessions over 16 weeks delivered by physiotherapists via telehealth (Zoom, USA). The first 4 sessions will be scheduled at approximately 2-week intervals, and the final 3 sessions will be scheduled at approximately 3-week intervals. Participants may be prescribed up to 1 h of home activities daily. The intervention progression follows a standard progression protocol, with mandatory advancement at each treatment session. Eligible graded motor imagery providers will be registered physiotherapists with experience treating CRPS who have completed a 2-day training course on graded motor imagery (delivered by training provider Noigroup, Australia). The study team will provide trial clinicians with 20 h of training on the graded motor imagery treatment protocol. Clinicians will be provided with a clinician manual and have access to clinical supervision during the trial.

Participants will receive resources that complement the delivery of the graded motor imagery intervention, including a CRPS pain education book (Complex Regional Pain Syndrome, an Explain Pain Handbook: Protectometer, Noigroup, Australia [developed for this trial, not currently for sale and attracts no royalties]), and access to an e-learning platform (Pathwright, USA), which is used to guide the home activities and direct the weekly intervention schedule. For the implicit motor imagery and mirror therapy activities, participants will receive the commercially available Recognise App, Recognise Flashcards, and a Mirror Box (Noigroup, Australia). The graded motor imagery intervention session schedule is provided in Supplementary Fig. 1. A detailed intervention description will be provided in a separate publication of the intervention program theory.

### Criteria for discontinuing or modifying allocated interventions {11b}

#### Memantine/placebo

The maximum tolerated dose (up to 40 mg/day) will be determined by the participant in consultation with the blinded trial physician and considers the participant’s willingness to continue the drug therapy despite adverse effects. Should participants need to temporarily suspend treatment due to intolerable adverse effects, they will be instructed to cease the medication for 48 h, at which point they will be reviewed by the trial physician. If the intolerable adverse effects have resolved, the participant will be instructed to resume the study medication at the highest tolerated dose before the onset of intolerable adverse effects. If the intolerable adverse effects have not resolved, the participant will be instructed to continue witholding the medication, with review by the trial physician every 48 h. The memantine/placebo therapy may be discontinued at the participant’s or physician’s discretion.

#### Graded motor imagery

The trial clinicians will individually tailor the graded motor imagery intervention for the study participants based on their symptoms, conceptual understanding of educational content, time, and preferences. Clinicians will determine the dose of each intervention component at each session. The graded motor imagery therapy may be discontinued at the participants’ discretion.

### Strategies to improve adherence to interventions {11c}

Adherence to memantine/placebo intervention will be measured via daily self-report medication diaries and pill counting in returned medication kits. Research staff will monitor medication diaries and contact participants who are not taking the study medicines as required to encourage adherence. Adherence to the graded motor imagery intervention will be measured by session attendance and daily self-report therapy diaries. Research staff and trial clinicians will monitor the number of completed graded motor imagery sessions and encourage attendance for participants who miss sessions. Trial clinicians will monitor the amount of home activities done through the e-learning portal and use communication skills to improve adherence when needed.

### Relevant concomitant care permitted or prohibited during the trial {11d}

Participants will be able to continue their usual treatment for CRPS which may include, but is not limited to, pharmacological, psychological, and rehabilitative treatments, excluding those listed in the exclusion criteria. We expect the concomitant use of therapies to be similar across all groups at baseline due to the randomised design of this study. Participants will record all concomitant therapies (and their dose) in healthcare use forms. Participants will be asked not to initiate new treatments during the trial therapy period, but should they do so, they will be asked to inform the study team and record these in the healthcare use forms. Participants will be asked not to access memantine or graded motor imagery outside of the trial.

### Provisions for post-trial care {30}

There will be no provisions for ancillary and post-trial care. Participants wishing to continue or recommence the trial therapies will need to consult their usual treating clinicians. The sponsor, Neuroscience Research Australia, adheres to the principles outlined in the Medicines Australia Guidelines for Compensation for Injury Resulting from Participation in a Company-Sponsored Clinical Trial. Neuroscience Research Australia has a ‘no fault’ compensation policy from UniMutual Clinical trials Protection insurance to the value of AUD$30M. The policy covers bodily injury to any research subject in a clinical trial.

### Outcomes {12}

The study outcome domains comprise those included in the core outcome measurement set for CRPS clinical studies [37]. The outcome measures were selected through consultation with the study’s consumer panel of people with lived experience of CRPS.

#### Primary outcomes

The dual primary outcomes are pain intensity and pain interference. These outcomes were selected by the study consumer panel as they were determined to represent the most important aspects of improvement for CRPS. Pain intensity is the average pain intensity of the affected limb over the previous 7 days assessed using the 11-point numeric rating scale (NRS) [38], with scores of 0 indicating ‘no pain’ and scores of 10 indicating ‘worst imaginable pain’. This scale has demonstrated validity and reliability for use in chronic pain research [39]. Pain interference is assessed using the Patient-Reported Outcomes Measurement Information System (PROMIS) Pain Interference v1.1 [40], measured over the previous 7 days, with higher scores indicating greater pain interference. This measure has demonstrated reliability and validity across different pain populations [40, 41]. Both dual primary outcomes will be assessed as differences in mean final values at 16 weeks post-randomisation.

#### Secondary outcomes

- Pain intensity, assessed over the previous 7 days using the 11-point NRS. Collected at 26 and 52 weeks post-randomisation.
- Pain interference, assessed over the previous 7 days using PROMIS Pain Interference v1.1. Collected at 26 and 52 weeks post-randomisation.
- Physical function, assessed at present using PROMIS Physical Function v2.0 [42], with higher scores indicating greater physical function. Collected at 16, 26, and 52 weeks post-randomisation.
- Fatigue, assessed over the previous 7 days using PROMIS Fatigue v1.0 [43], with higher scores indicating greater fatigue. Collected at 16, 26, and 52 weeks post-randomisation.
- Self-efficacy to manage symptoms, assessed at present using PROMIS Self-Efficacy Manage Symptoms v1.0 [44], with higher scores indicating greater self-efficacy to manage symptoms. Collected at 16, 26, and 52 weeks post-randomisation.
- Cognitive function, assessed over the previous 7 days using PROMIS Cognitive Function Abilities Subset v2.0 [45], with higher scores indicating greater cognitive function. Collected at 16, 26, and 52 weeks post-randomisation.
- Depressive symptoms, assessed over the previous 7 days using PROMIS Depression v1.0 [46], with higher scores indicating greater depressive symptoms. Collected at 16, 26, and 52 weeks post-randomisation.
- Health-related quality of life, assessed over the previous 7 days and ‘generally’ using PROMIS Global Health short form v1.1 [47], with higher scores indicating better health-related quality of life. Collected at 16 and 52 weeks post-randomisation.
- Pain self-efficacy, assessed at present using Pain Self-Efficacy Questionnaire (two-item short form) (PSEQ-2) [48]. The PSEQ-2 comprises two 7-point Likert scales (total score = 14), with higher scores indicating higher pain self-efficacy. Collected at 16, 26, and 52 weeks post-randomisation.
- Patient Global Impression of Change [37], assessed at present using a 7-point scale, with higher scores indicating greater improvements. Collected at 16 weeks post-randomisation.
- CRPS severity, assessed at present using the CRPS Severity Score [49, 50]. The CRPS Severity Score assesses 8 self-report symptoms and 8 clinician-assessed signs (total score = 16), with higher scores indicating greater CRPS severity. Collected at 16 weeks post-randomisation.
- Cost effectiveness, assessed as prescription and over-the-counter medicines use and visits (yes/no) to a healthcare provider for CRPS. Collected via self-report questionnaires and MBS and PBS data at 16, 26, and 52 weeks post-randomisation.
- Adverse events, assessed as the number of participants with at least one adverse event, serious adverse event, or specific adverse event (yes/no). Collected during treatment and during follow-up.

Continuous outcomes will be assessed as differences in mean final values at each timepoint; binary outcomes will be assessed as ratios of proportions.

PROMIS instruments use a *T*-score metric, in which 50 is the mean of the reference population and 10 is the standard deviation of that population [51]. All PROMIS scales, except for Global Health short form v1.1, will be measured using computer adaptive tests. Computer adaptive tests use item response theory to provide precise measurement using few items, and allow for scores that are directly comparable across different selections of items [52].

Pain intensity, pain interference, physical function, self-efficacy, and cognitive function will also be measured at 12 weeks to explore the mechanisms by which the graded motor imagery intervention exerts its effects. To evaluate the effect of adhering to the interventions, the following outcomes will also be measured: weekly measures of pain intensity, item 4 of the Credibility/Expectancy Questionnaire [53], Medical Form Confidence (weekly); International Physical Activity Questionnaire Short Form [54] (end of treatment only). See Methods for additional analyses (e.g. subgroup analyses) {20b} for further information.

### Participant timeline {13}

See Table 1.

**Table 1:**
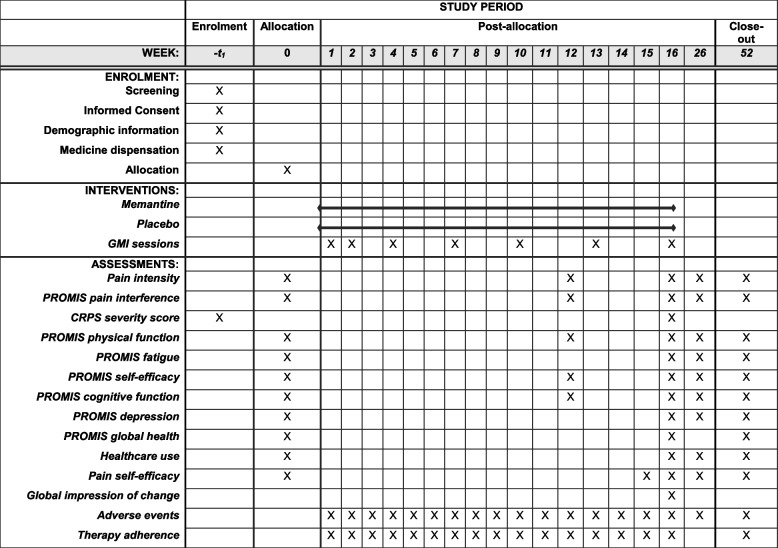
Schedule of study procedures, outcomes, and visit windows

*GMI* Graded motor imagery

### Sample size {14}

The sample size calculation was based on a factorial analysis, where interventions are assumed to have independent effects. For the primary outcome of pain intensity at 16 weeks, we will be guided by a between-group difference of 1 point on an 11-point NRS to justify implementation of the interventions as suggested for chronic pain RCTs [55]. For the primary outcome of pain interference at 16 weeks, we will be guided by a between-group difference *T*-score metric of 5 to justify implementation of the interventions.

Using a repeated measures model with outcomes measured at baseline and follow-up timepoints, and with a correlation between adjacent time points of 0.7 [56], 182 participants are required to provide 90% power with a two-sided 2.5% significance level to detect an effect (of memantine compared to placebo, or graded motor imagery compared to no graded motor imagery) of either 1 point on an 11-point NRS for pain intensity, assuming a standard deviation of 2, or 5 *T*-metric points on the PROMIS Pain Interference scale, assuming a standard deviation of 10. Allowing for 10% dropout, we will aim to recruit a total of 204 participants (rounded up from 200.2).

### Recruitment {15}

A decentralised design will be used to overcome barriers to participant recruitment common to CRPS trials, enabling nation-wide recruitment and increasing equity of access to all Australians, including those living in rural and remote geographical regions. We will use three strategies to recruit our target sample size: community-based advertisements using social media advertising (Facebook and Instagram); collaboration with primary and secondary care clinics; and collaboration with CRPS consumer and support groups. All potential participants will be directed to the study website (https://memoir.neura.edu.au/) where they will initiate eligibility screening.

## Assignment of interventions: allocation

### Sequence generation {16a}

The allocation sequence will be randomly generated a priori using Stata 14.0 software. Enrolled participants will be randomised to one of 4 intervention groups (1:1:1:1). The allocation sequence for memantine and placebo is a permuted block list consisting of blocks of randomly varying sizes, in equal proportion. The allocation sequence for the graded motor imagery and no graded motor imagery arms will be controlled by separate permuted block lists: one for memantine and one for placebo, each with blocks of randomly varying sizes, in equal proportion.

### Concealment mechanism {16b}

The pharmaceutical supply company (Eramol, UK) will be provided with the randomisation schedule. Eramol will package and label the study medication according to the randomisation schedule and seal the packages for transportation to the research pharmacy, Syntro (Melbourne, Australia). Each medication kit will be numbered randomly. A central web-based randomisation system (interface on Qualtrics software) will hold allocations to the graded motor imagery group. The blinding of the drug allocations provides additional concealment of the graded motor imagery allocation. These features will ensure that neither the person being randomised nor trial personnel are aware of the forthcoming allocation.

### Implementation {16c}

The allocation sequence will be generated by an independent statistician from the Australian National Health and Medical Research Council Clinical Trial Centre. The statistician responsible for the generation of the allocation sequence will not be involved in participant recruitment, treatment, data collection, or analysis. Only the National Health and Medical Research Council Clinical Trial Centre, drug manufacturer, and unblinded Data and Safety Monitoring Board (DSMB) statistician will hold the randomisation schedule. Trained research assistants will use the web-based randomisation system to inform eligible participants of their allocation to the graded motor imagery group.

## Assignment of interventions: blinding

### Who will be blinded {17a}

In this factorial trial, participants and study personnel will be blinded to memantine and placebo group assignment and unblinded to graded motor imagery assignment. As the CRPS Severity Score is the only clinician-assessed outcome, it will be evaluated by a clinician who is blinded to both treatment factors to minimise detection bias. All other outcomes are self-reported by participants and therefore unblinded for the graded motor imagery comparison. Blinding to memantine and placebo assignment will be maintained for the entire duration of the trial until all data have been collected and data analysis and interpretation have been completed. The independent statistician will be blinded to all intervention assignments until the preliminary analyses are complete (see General analysis principles).

### Procedure for unblinding if needed {17b}

To maintain trial integrity, blinded trial personnel will only be unblinded in exceptional circumstances (for example, when knowledge of the actual treatment received is essential for further management of the participant). Requests for unblinding will be considered by the chief investigator, where practical, in consultation with the chair of the DSMB. Unblinding will be recorded and reported as a deviation from protocol and will not result in study discontinuation.

## Data collection and management

### Plans for assessment and collection of outcomes {18a}

Study outcomes, measures (including references for reliability and validity), and timing of assessment are outlined in Outcomes {12}. Screening and demographic data will be entered by participants and trained research staff into REDCap. All outcome data, except for the CRPS severity score which requires input from a clinician, will be entered directly into REDCap by participants. Should outcome data be obtained via telephone, it will be entered into REDCap by a research assistant and checked by another staff member.

### Plans to promote participant retention and complete follow-up {18b}

Participant retention will be optimised through regular participant contact and check-in telephone calls. The importance of completing follow-up assessments will be emphasised. Automated email reminders will be sent via REDCap if outcome assessments are not completed within 24 h of their delivery. If outcome measures remain incomplete following reminders, the study team will contact participants via SMS or telephone.

Participants have the right to withdraw from the study at any time. Where possible, a reason for withdrawal will be recorded. If participants express that they wish to discontinue the trial treatment(s), the study team will ascertain whether the participant is willing to continue to complete outcome assessments. If participants wish to withdraw from future outcome assessments, all data collected up until the moment of withdrawal will be retained unless objections are made by the participant.

### Data management {19}

All participant data, except for MBS/PBS data, will be collected, managed, and stored on the web-based REDCap platform. REDCap uses intuitive electronic case report forms, pre-defined fields, and real-time data validation and integrity checks to ensure data consistency and quality. REDCap provides encryption between the data entry client and the server and individual access to the database via secure login with 2-factor authentication. The REDCap database will only be accessible by approved research staff.

The REDCap application and data will be housed on a server managed by the University of New South Wales Sydney. The database is securely backed up as per standard University of New South Wales procedures for data security. For the purpose of specialised autoscoring and functionality of the PROMIS computerised adaptive testing, REDCap communicates with Vanderbilt University’s server (Nashville, TN, USA). The security of this server is ensured, and no personally identifiable information is sent to the server. Participants are informed of this requirement before they provide informed consent.

The MEMOIR study data will be archived for 15 years, consistent with clinical trial recommendations outlined in the National Health and Medical Research Council’s ‘Australian Code for the Responsible Conduct of Research’, after which electronic information will be permanently deleted.

### Confidentiality {27}

All participants will be provided with an identification number. All data will be coded with this number so that any data collected will only be identifiable by the code. All analyses will be done using de-identified data. Results will be disseminated using aggregate data to ensure confidentiality is preserved.

### Plans for collection, laboratory evaluation, and storage of biological specimens for genetic or molecular analysis in this trial/future use {33}

Biological specimens will not be collected in this study.

## Statistical methods

### General analysis principles

The primary objective of the trial is to estimate the effects of memantine compared to placebo memantine and graded motor imagery compared to no graded motor imagery on pain intensity and pain interference. Consequently, the focus of the analysis and the interpretation of the trial findings will be on the magnitude of point estimates of effects and the precision of those estimates, quantified with confidence intervals. The analysis will be supplemented by *p* values but not by claims about statistical significance or non-significance [57].

Preliminary, blinded analyses will be conducted before the final analysis. The preliminary analyses will be conducted using randomly permuted ‘dummy’ treatment allocations. These analyses will identify any issues with the analysis pipeline and enable decisions to be made about, for example, distributional assumptions, without knowledge of the implications of those decisions on the trial’s findings. Once those issues have been resolved and the statistical code has been finalised, the definitive analysis will be conducted using true allocations. Analyses will be performed using R and Stata software.

### Descriptive analysis

A Consolidated Standards of Reporting Trial (CONSORT) diagram will be used to summarise the participant flow through the MEMOIR trial [58] (Supplementary Fig. 2). The diagram will show the number of participants assessed for eligibility and excluded (with reasons), and for each comparison, the number randomised, allocated to (and received) interventions, discontinued interventions (with reasons), lost to follow-up for the dual primary outcomes (for each timepoint), analysed in the primary analysis, and excluded from analysis (with reasons).

Baseline demographic and clinical data will be presented to characterise the study sample. Counts and percentages will be presented for binary and categorical variables. Means and standard deviations or medians and interquartile ranges will be presented for continuous variables. The following characteristics will be described, grouped as per the main comparisons (memantine versus placebo, graded motor imagery versus no graded motor imagery) (see Supplementary Table 1):

- Age
- Sex
- Body mass index
- Smoker status
- Weekly alcohol consumption
- Ethnicity
- Aboriginal or Torres Strait Islander
- Geographical remoteness
- Index of relative socioeconomic advantage and disadvantage
- Work status
- Education level
- CRPS duration
- Inciting event
- Primary CRPS location
- Limb dominance before CRPS
- Compensability of CRPS
- Prescription medicine use
- Over-the-counter-medicine use
- CRPS healthcare visits

### Statistical methods for primary and secondary outcomes {20a}

#### Main analysis

All outcomes will be analysed according to the intention to treat principle. The analysis will involve estimation of the marginal mean effect of memantine compared to placebo and the marginal mean effect of graded motor imagery compared to no graded motor imagery on pain intensity and pain interference at 16 weeks. Additional information on the estimands of interest, including handling of intercurrent events, is provided in Table 2. As the trial has dual primary outcomes, interval estimates will be 97.5% confidence intervals. There will be no multiplicity adjustment for the two interventions as the factorial design answers two distinct questions.

**Table 2:** Trial estimands

Estimand component	Definition
Interventions to be compared	To estimate the average causal effect of memantine, we will compare the mean outcome of trial participants allocated to receive memantine and graded motor imagery or memantine only with the mean outcome of trial participants allocated to receive placebo and graded motor imagery or placebo only To estimate the average causal effect of graded motor imagery, we will compare the mean outcome of trial participants allocated to receive memantine and graded motor imagery or placebo and graded motor imagery with the mean outcome of participants allocated to receive memantine only or placebo only
Population of interest	Adults with CRPS as defined by the trial eligibility criteria
Outcome of interest	Pain intensity, measured on a 11-point NRS; pain interference, measured with PROMIS pain interference v1.1
Summary measure (population-level)	Mean difference
Intercurrent events	All intercurrent events relating to memantine and graded motor imagery (e.g. intervention discontinuation, use of non-trial treatments) will be handled using a treatment policy strategy

For each of the dual primary outcomes, we will fit a longitudinal mixed-effects model that includes terms for the fixed effects of each intervention, the three follow-up timepoints, the intervention by time interactions, the two-way interaction between the interventions, and the baseline values of the outcome variables. In addition, the models will include terms for the fixed effects of the duration of CRPS (modelled as linear or linear with log transformation) and workplace compensation status, as well as random intercepts for trial participants.

The coefficients in the model are conditional on the other factors in the model. However, our primary interest is in the estimation of the marginal effects of the interventions. Point and interval estimates of the marginal effect of each intervention at 16 weeks will be obtained by appropriately weighting the sum of the relevant model coefficients and the elements of the variance-covariance matrix using the -margins- command in Stata. Secondary outcomes and safety outcomes (the number of participants experiencing at least one adverse event, specific adverse events, serious adverse events) will be analysed in the same way except that log binary models will be used for binary outcomes. For each comparison, mean differences or risk ratios, each with 97.5% confidence intervals, will be presented in tables (see Supplementary Tables 2, 3, 4, and 5).

### Interim analyses {21b}

A formal interim analysis is not planned. The DSMB will use clinical expertise to perform an unblinded review of accruing safety data (adverse events and serious adverse events) at regular intervals and advise the Steering Committee whether the trial should be stopped for safety or ethical concerns.

### Methods for additional analyses (e.g. subgroup analyses) {20b}

#### Sensitivity analyses

Sensitivity analyses will be conducted to assess the impact of deviations to the ‘no interaction’ assumption on the primary outcomes. As recommended by Kahan et al. [59], the size, confidence intervals, and *p* value of the treatment-by-treatment interaction will be inspected to assess the plausibility of the assumption of no interaction underpinning the interpretation of the marginal estimator. Further sensitivity analyses will be conducted on the primary outcomes to estimate the effects of each intervention conditional on the presence or absence of the other intervention, again using the -margins- command in Stata. The effect of each intervention combination (memantine and graded motor imagery; memantine only; graded motor imagery and placebo) compared with the double control group (placebo only) will also be estimated (Supplementary Table 6). Multi-arm analyses remain unbiased even when interventions interact [59].

The analyses described below may be reported in secondary publications.

#### Subgroup analyses

Effect modification will be assessed by adding the following variables (one at a time, along with their interactions with one intervention at all time points) to the main analysis model, for each of the dual primary outcomes:

- Sex (male/female)
- Workplace compensation status (yes/no)
- CRPS duration (months; continuous)
- Opioid use (yes/no)

#### Cost effectiveness analysis

A within-trial economic evaluation will be conducted to assess the cost-effectiveness of memantine compared with placebo and graded motor imagery compared with no graded motor imagery, from a health care and societal perspective. Health outcomes will include clinical endpoints and health-related quality of life measured using the PROMIS Global Health. Utility values will be derived using the PROMIS-Preference (PROPr) Scoring System, which maps PROMIS responses onto a preference-based utility scale [60]. These utility values will be used to estimate quality-adjusted life years (QALYs) gained. Health care resource use will be estimated using linked administration data (MBS and PBS data) and patient questionnaires to capture the cost of health care utilisation, medication use, and out-of-pocket costs. Results will be reported as the cost per QALY gained and uncertainty explored using non-parametric bootstrapping. Sensitivity analyses will be conducted, where feasible, to test the robustness of the results. If the trial does not demonstrate a statistically significant difference in effectiveness, alternative economic evaluation approaches will be considered. The economic evaluation will follow the Consolidated Health Economic Evaluation Reporting Standards (CHEERS) guideline [61].

#### Mediation analysis

A causal mediation analysis will be performed to guide graded motor imagery optimisation and implementation. This analysis will estimate the effects of graded motor imagery on pain intensity and pain interference through the putative mediators [62].

#### Complier average causal effect

If there is significant non-adherence with the allocated interventions, we will estimate the complier-average causal effect (CACE) using instrumental variable estimation [63, 64]. For the purposes of the CACE analysis, we will define memantine adherence as taking 80% or more of the prescribed dose (self-reported), and graded motor imagery adherence as attendance at 5 or more of 7 sessions.

### Methods in analysis to handle protocol non-adherence and any statistical methods to handle missing data {20c}

The analyses are based on the intention-to-treat principle, where all participants will be assessed in the groups to which they are randomised, regardless of the intervention received or intervention adherence. A summary of the interventions allocated and received will be reported for each comparison. Details on the presentation of intervention adherence and protocol deviations are provided in Appendices 1 and 2 and Supplementary Tables 7 and 8.

Efforts will be taken to minimise the amount of missing outcome data (see Plans to promote participant retention and complete follow-up {18b}). For each outcome, the amount of missing data will be reported. The nature and pattern of data missingness will be explored to determine, as far as is possible, whether data can be treated as missing completely at random (MCAR) or missing at random (MAR). If more than 10% of the primary outcome data are missing and the MAR assumption appears plausible, a sensitivity analysis will be conducted in which the main analysis is replicated using multiple imputation [65, 66].

### Plans to give access to the full protocol, participant-level data, and statistical code {31c}

A data sharing plan will be established in accordance with Australian New Zealand Clinical Trials Registry requirements. This includes provisions for sharing de-identified participant-level data, the full trial protocol, participant information forms, ethical approvals, and analytic code, subject to participant consent and regulatory requirements. Requests for de-identified participant-level data or analytic code will be reviewed to ensure they align with the study’s data use agreements.

## Oversight and monitoring

### Composition of the coordinating centre and trial steering committee {5d}

MEMOIR is sponsored by Neuroscience Research Australia. The coordinating study centre at Neuroscience Research Australia will perform all recruitment, screening, and monitoring activities. A dedicated trial manager will oversee all aspects of daily trial conduct, assist with governance and regulatory matters, and provide quality control. A Trial Management Committee, comprising the chief investigator, trial manager, and research assistants, will meet monthly to ensure the project delivers its objectives, and monitors the project’s budget, schedule, milestones, and risks.

A Trial Steering Committee will be formed, comprising scientific experts with content and methodological expertise, medical training, and consumers. Chair and co-chair positions will be appointed. The Trial Steering Committee will be responsible for the general oversight of the study, providing scientific advice regarding all aspects of the study design, protocol development, conduct, and data collection. The Trial Steering Committee will develop, approve, and maintain the Trial Steering Committee Charter. The MEMOIR Trial Steering Committee will be the decision-making body responsible for implementing modifications to the protocol, including those that result from recommendations from the study’s DSMB. Trial Steering Committee meetings will take place every 6 months.

### Patient and public involvement and engagement

A consumer panel was formed prior to the commencement of the study. The panel includes people who have or have recovered from CRPS and consumer advocates. The panel is responsible for the provision of participant-relevant recommendations for aspects of the trial including development and conduct, quality control, and community engagement. The MEMOIR investigators provided external consumer training to all members. Over a series of focus groups, the panel informed critical features of the study including the research question, the acceptability of the screening procedures and trial interventions, and the selection of primary and secondary outcomes. During the conduct of the trial, the panel will oversee study processes and review media briefs to ensure appropriate content and language. The panel will be involved in the interpretation and dissemination of study results and will develop a lay language summary of trial results. The consumer contributions to the study will be reported in the published manuscript. A separate group of five people with lived experience of CRPS was involved in the development of the CRPS Handbook. This involvement included initial brainstorming about illustrations and the use of metaphors, iterative review of text, and final review of combined text and illustrations. Data from other people diagnosed with CRPS, presented in full elsewhere [67], were also integrated into that trial-specific patient handbook.

### Composition of the data monitoring committee, its role and reporting structure {21a}

A DSMB will be appointed to review the safety and progress of the trial. The DSMB will comprise a chair and three members with clinical or statistical expertise, all independent from the sponsor and competing interests. The DSMB will review accumulating unblinded safety data (intervention withdrawals, adverse events, and serious adverse events) at regular intervals and advise the Trial Steering Committee Chair if any change to the study is recommended. The DSMB reports directly to the Trial Steering Committee Chair, who liaises with the Trial Steering Committee on any recommendations.

### Adverse event reporting and harms {22}

Safety monitoring and reporting will be conducted in accordance with the NHMRC guidance on Safety Monitoring and Reporting in Clinical Trials Involving Therapeutic Goods and the International Council for Harmonisation Good Clinical Practice guidelines [36, 68]. Adverse events will be collected by active capture (weekly during treatment [weeks 1 to 16] and during follow-up [week 26, week 52]), or by passive capture (participant report via telephone, email, or SMS). Data on adverse effects specific to memantine (those listed on the memantine product information), new medical conditions, or the worsening of existing medical conditions will be collected. Each adverse event reported will be assessed for:

- Seriousness: any medical occurrence that results in death; is life-threatening; requires inpatient hospitalisation or prolongation of an existing hospitalisation; results in persistent or significant disability/incapacity; is a congenital anomaly or birth defect; or is medically significant or important event or reaction
- Causality: whether the event has a reasonable causal relationship to the trial intervention
- Expectedness: whether the event is listed in the memantine product information
- Severity: mild/moderate/severe

The DSMB will periodically review overall safety data to determine trends of events or identify safety issues that would not be apparent on an individual case basis. A safety management plan will outline the flow and timelines for reporting all relevant safety information between the coordinating centre, the sponsor, the Human Research Ethics Committee, and the Australian Therapeutic Goods Administration.

### Frequency and plans for auditing trial conduct {23}

All trial procedures are outlined in study manuals to ensure compliance with the protocol and ICH Good Clinical Practice guidelines. Audits will be conducted by a staff member at the advice of the Steering Committee and will concern enrolment, consent, eligibility, adherence to trial interventions, policies to protect participants (including reporting of harm), and completeness, accuracy, and timeliness of data collection. An external audit, independent from the investigators and the sponsor, will be conducted before commencing the trial and during its conduct.

### Plans for communicating important protocol amendments to relevant parties (e.g. trial participants, ethical committees) {25}

Changes and amendments to the protocol will be reviewed and approved by the Trial Steering Committee and investigators. Approval of amendments by the Human Research Ethics Committee is required prior to implementation. Significant protocol changes will be posted to the Australian New Zealand Clinical Trials Registry and reported to the Data Safety Monitoring Board.

### Dissemination plans {31a}

On completion of the trial, the data will be analysed and a final report will be prepared in accordance with the CONSORT reporting guideline [58], including extensions for factorial trials [29] and the Template for Intervention Description and Replication (TIDieR) [69]. Authorship eligibility for study publications will be determined in accordance with the criteria outlined by the International Committee of Medical Journal Editors. The final report will be submitted for publication in a peer-reviewed scientific journal and presented at national and international conferences. Results may also be presented to the media, general public, and relevant policy bodies. There are no publication restrictions, and professional writers will not be involved in the preparation of the manuscript. A one-page common language summary of the study findings will be sent to all participants and shared with CRPS patient support groups.

## Discussion

CRPS is a debilitating pain disorder with considerable personal, societal, and economic impacts. Our patient advisory group reported that CRPS patients want effective, low-cost, pharmacological and non-pharmacological interventions that can be received at home, to reduce CRPS pain intensity and pain interference. There are no interventions for CRPS, supported by high- or moderate-certainty evidence, that meet this need. We identified two promising interventions in pilot studies—memantine and graded motor imagery—but their effects are yet to be definitively evaluated in adequately powered, high-quality clinical trials. The MEMOIR trial will test, for the first time, the efficacy of memantine, and the effectiveness of graded motor imagery, on pain intensity and pain interference for people with CRPS, in the largest academic CRPS trial to date.

Our previous research showed that CRPS trials are underpowered, lack diverse study samples, and involve short follow-up [15]. The MEMOIR trial aims to overcome these critical limitations by using a decentralised, 2 × 2 factorial design. Decentralised recruitment enables enrolment of patients where recruitment would usually be subject to geographical and socioeconomic constraints, strengthening the trial’s external validity and ensuring that potential research benefits are equitably distributed across all population segments [70, 71]. The 2 × 2 factorial design permits a ‘2-in-1’ evaluation of two interventions in a single trial without increasing the sample size [29]. Factorial trials have been proposed as one method to maximise efficiency for rare conditions that are challenged by participant recruitment [72, 73].

The MEMOIR trial will provide robust evidence to guide international management of CRPS. It will provide novel information on a repurposed medicine with potential analgesic benefits and address the critical clinical uncertainty regarding the effectiveness of graded motor imagery. If shown to be effective, both interventions are low-cost and can be delivered in clinics or direct to patients' homes.

## Trial status

This document includes version 8.0 of the protocol and the statistical analysis plan. Participant recruitment began on 12 May 2021 and is projected to be completed by Q2 2026. All outcomes will be analysed collectively at the completion of the 52-week follow-up for the last randomised participant. The anticipated date of the final assessment is Q2 2027.

## Supplementary Information

Supplementary Material 1.

## Data Availability

Participants may specifically request a copy of their data from the chief investigator; these data will be provided after publication of the final trial report. The de-identified trial data of consenting participants may be shared with other researchers for use in future projects, provided the project has received separate ethics approval from a Human Research Ethics Committee.

## Abbreviations

CONSORT: Consolidated Standards of Reporting Trials
CRPS: Complex regional pain syndrome
DSMB: Data Safety Monitoring Board
IASP: International Association for the Study of Pain
MBS: Medicare Benefits Schedule
NMDA: N-methyl D-aspartate
NRS: Numeric rating scale
PROMIS: Patient-Reported Outcomes Measurement Information System
PBS: Pharmaceutical Benefits Scheme
