# Supplementary material for: Memantine and graded motor imagery for complex regional pain syndrome (MEMOIR): study protocol and statistical analysis plan for a decentralised, 2 × 2 factorial randomised trial
Source: Trials. 2025 Dec 29;27:107. doi: 10.1186/s13063-025-09383-8 (PMC12874714; doi:10.1186/s13063-025-09383-8)
Supplement: Supplementary file 1 — Supplementary Material 1. [file 13063_2025_9383_MOESM1_ESM.docx]

**Supplementary Figure 1. Graded motor imagery session schedule.**

Graded motor imagery session schedule. Dark colour shades denote component must be done at indicated session; light colour shades denote flexible start/finish times for each element for fast or slow progressors, respectively.

**Supplementary Figure 2: CONSORT Flowchart**

CRPS: complex regional pain syndrome; IASP: International Association for the Study of Pain

**Supplementary Table 1: Baseline characteristics**

|  | **Comparison 1** | | **Comparison 2** | |
| --- | --- | --- | --- | --- |
|  | **Memantine (n=)** | **Placebo (n=)** | **Graded motor imagery (n=)** | **No graded motor imagery (n =)** |
| Age, years | *xx.x (SD), n* | *xx.x (SD), n* | *xx.x (SD), n* | *xx.x (SD), n* |
| Sex |  |  |  |  |
| Male | *n/N (%)* | *n/N (%)* | *n/N (%)* | *n/N (%)* |
| Female | *n/N (%)* | *n/N (%)* | *n/N (%)* | *n/N (%)* |
| Body mass index | *xx.x (SD), n* | *xx.x (SD), n* | *xx.x (SD), n* | *xx.x (SD), n* |
| Current smoker | *n/N (%)* | *n/N (%)* | *n/N (%)* | *n/N (%)* |
| Alcohol consumption |  |  |  |  |
| <10 standard drinks weekly | *n/N (%)* | *n/N (%)* | *n/N (%)* | *n/N (%)* |
| >10 standard drinks weekly | *n/N (%)* | *n/N (%)* | *n/N (%)* | *n/N (%)* |
| Ethnicity |  |  |  |  |
| Oceanian | *n/N (%)* | *n/N (%)* | *n/N (%)* | *n/N (%)* |
| Non-West European | *n/N (%)* | *n/N (%)* | *n/N (%)* | *n/N (%)* |
| Southern and Eastern European | *n/N (%)* | *n/N (%)* | *n/N (%)* | *n/N (%)* |
| North African and Middle Eastern | *n/N (%)* | *n/N (%)* | *n/N (%)* | *n/N (%)* |
| South-East Asian | *n/N (%)* | *n/N (%)* | *n/N (%)* | *n/N (%)* |
| North-East Asian | *n/N (%)* | *n/N (%)* | *n/N (%)* | *n/N (%)* |
| Southern and Central Asian | *n/N (%)* | *n/N (%)* | *n/N (%)* | *n/N (%)* |
| People of the Americas | *n/N (%)* | *n/N (%)* | *n/N (%)* | *n/N (%)* |
| Sub-Saharan African | *n/N (%)* | *n/N (%)* | *n/N (%)* | *n/N (%)* |
| Aboriginal or Torres Strait Islander | *n/N (%)* | *n/N (%)* | *n/N (%)* | *n/N (%)* |
| Modified Monash level |  |  |  |  |
| 1 (metropolitan) | *n/N (%)* | *n/N (%)* | *n/N (%)* | *n/N (%)* |
| 2 (regional centres) | *n/N (%)* | *n/N (%)* | *n/N (%)* | *n/N (%)* |
| 3 (large rural towns) | *n/N (%)* | *n/N (%)* | *n/N (%)* | *n/N (%)* |
| 4 (medium rural towns) | *n/N (%)* | *n/N (%)* | *n/N (%)* | *n/N (%)* |
| 5 (small rural towns) | *n/N (%)* | *n/N (%)* | *n/N (%)* | *n/N (%)* |
| 6 (remote communities) | *n/N (%)* | *n/N (%)* | *n/N (%)* | *n/N (%)* |
| 7 (very remote communities) | *n/N (%)* | *n/N (%)* | *n/N (%)* | *n/N (%)* |
| SEIFA IRSAD decile |  |  |  |  |
| 1 (most disadvantaged) | *n/N (%)* | *n/N (%)* | *n/N (%)* | *n/N (%)* |
| 2 | *n/N (%)* | *n/N (%)* | *n/N (%)* | *n/N (%)* |
| 3 | *n/N (%)* | *n/N (%)* | *n/N (%)* | *n/N (%)* |
| 4 | *n/N (%)* | *n/N (%)* | *n/N (%)* | *n/N (%)* |
| 5 | *n/N (%)* | *n/N (%)* | *n/N (%)* | *n/N (%)* |
| 6 | *n/N (%)* | *n/N (%)* | *n/N (%)* | *n/N (%)* |
| 7 | *n/N (%)* | *n/N (%)* | *n/N (%)* | *n/N (%)* |
| 8 | *n/N (%)* | *n/N (%)* | *n/N (%)* | *n/N (%)* |
| 9 | *n/N (%)* | *n/N (%)* | *n/N (%)* | *n/N (%)* |
| 10 (most advantaged) | *n/N (%)* | *n/N (%)* | *n/N (%)* | *n/N (%)* |
| Work status |  | | | |
| Full-time employment | *n/N (%)* | *n/N (%)* | *n/N (%)* | *n/N (%)* |
| Part-time employment | *n/N (%)* | *n/N (%)* | *n/N (%)* | *n/N (%)* |
| Full-time student | *n/N (%)* | *n/N (%)* | *n/N (%)* | *n/N (%)* |
| Unemployed | *n/N (%)* | *n/N (%)* | *n/N (%)* | *n/N (%)* |
| Not working due to CRPS | *n/N (%)* | *n/N (%)* | *n/N (%)* | *n/N (%)* |
| Education level |  |  |  |  |
| Year 10 (School certificate) | *n/N (%)* | *n/N (%)* | *n/N (%)* | *n/N (%)* |
| Vocational certificate | *n/N (%)* | *n/N (%)* | *n/N (%)* | *n/N (%)* |
| Year 12 (HSC) | *n/N (%)* | *n/N (%)* | *n/N (%)* | *n/N (%)* |
| Diploma | *n/N (%)* | *n/N (%)* | *n/N (%)* | *n/N (%)* |
| Bachelor degree or higher | *n/N (%)* | *n/N (%)* | *n/N (%)* | *n/N (%)* |
| CRPS duration (months) | *xx.x (SD), n* | *xx.x (SD), n* | *xx.x (SD), n* | *xx.x (SD), n* |
| Inciting injury |  |  |  |  |
| Fracture | *n/N (%)* | *n/N (%)* | *n/N (%)* | *n/N (%)* |
| Surgery | *n/N (%)* | *n/N (%)* | *n/N (%)* | *n/N (%)* |
| Sprain | *n/N (%)* | *n/N (%)* | *n/N (%)* | *n/N (%)* |
| Trauma | *n/N (%)* | *n/N (%)* | *n/N (%)* | *n/N (%)* |
| Other | *n/N (%)* | *n/N (%)* | *n/N (%)* | *n/N (%)* |
| Spontaneous | *n/N (%)* | *n/N (%)* | *n/N (%)* | *n/N (%)* |
| Primary CRPS location |  |  |  |  |
| Hand/wrist | *n/N (%)* | *n/N (%)* | *n/N (%)* | *n/N (%)* |
| Elbow | *n/N (%)* | *n/N (%)* | *n/N (%)* | *n/N (%)* |
| Upper arm | *n/N (%)* | *n/N (%)* | *n/N (%)* | *n/N (%)* |
| Foot/ankle | *n/N (%)* | *n/N (%)* | *n/N (%)* | *n/N (%)* |
| Knee | *n/N (%)* | *n/N (%)* | *n/N (%)* | *n/N (%)* |
| Limb dominance before CRPS |  |  |  |  |
| Affected side | *n/N (%)* | *n/N (%)* | *n/N (%)* | *n/N (%)* |
| Non-affected side | *n/N (%)* | *n/N (%)* | *n/N (%)* | *n/N (%)* |
| CRPS compensable | *n/N (%)* | *n/N (%)* | *n/N (%)* | *n/N (%)* |
| Prescription medicine use |  |  |  |  |
| ≥1 prescription medicine | *n/N (%)* | *n/N (%)* | *n/N (%)* | *n/N (%)* |
| Strong opioids | *n/N (%)* | *n/N (%)* | *n/N (%)* | *n/N (%)* |
| Weak opioids | *n/N (%)* | *n/N (%)* | *n/N (%)* | *n/N (%)* |
| Anticonvulsants | *n/N (%)* | *n/N (%)* | *n/N (%)* | *n/N (%)* |
| Antidepressants | *n/N (%)* | *n/N (%)* | *n/N (%)* | *n/N (%)* |
| Corticosteroids | *n/N (%)* | *n/N (%)* | *n/N (%)* | *n/N (%)* |
| NSAIDs | *n/N (%)* | *n/N (%)* | *n/N (%)* | *n/N (%)* |
| Cannabinoids |  |  |  |  |
| Over-the-counter medicine use |  |  |  |  |
| ≥1 over-the-counter medicine | *n/N (%)* | *n/N (%)* | *n/N (%)* | *n/N (%)* |
| Paracetamol | *n/N (%)* | *n/N (%)* | *n/N (%)* | *n/N (%)* |
| NSAIDs | *n/N (%)* | *n/N (%)* | *n/N (%)* | *n/N (%)* |
| Topicals | *n/N (%)* | *n/N (%)* | *n/N (%)* | *n/N (%)* |
| Healthcare visits in past 6 months |  |  |  |  |
| ≥1 health provider visit | *n/N (%)* | *n/N (%)* | *n/N (%)* | *n/N (%)* |
| GP | *n/N (%)* | *n/N (%)* | *n/N (%)* | *n/N (%)* |
| Pain specialist | *n/N (%)* | *n/N (%)* | *n/N (%)* | *n/N (%)* |
| Psychiatrist | *n/N (%)* | *n/N (%)* | *n/N (%)* | *n/N (%)* |
| Psychologist | *n/N (%)* | *n/N (%)* | *n/N (%)* | *n/N (%)* |
| Other medical specialist | *n/N (%)* | *n/N (%)* | *n/N (%)* | *n/N (%)* |
| Occupational therapist | *n/N (%)* | *n/N (%)* | *n/N (%)* | *n/N (%)* |
| Physiotherapist | *n/N (%)* | *n/N (%)* | *n/N (%)* | *n/N (%)* |

CRPS: complex regional pain syndrome; NSAID: non-steroidal anti-inflammatory drug. SEIFA IRSAD: socio-economic indexes for areas index of relative socio-economic advantage and disadvantage.

*Allied health professional, alternative medicine

**Supplementary table 2: Marginal effects of interventions on primary and secondary outcomes**

|  | **Memantine** | **Placebo** | **Mean difference** | **p value** | **Graded motor imagery** | **No graded motor imagery** | **Mean difference** | **p value** |
| --- | --- | --- | --- | --- | --- | --- | --- | --- |
|  | **Mean (SD)** | **Mean (SD)** |  |  | **Mean (SD)** | **Mean (SD)** |  |  |
| **Pain intensity** | | | | | | | | |
| Baseline | *x.x (x.x), n* | *x.x (x.x), n* |  |  | *x.x (x.x), n* | *x.x (x.x), n* |  |  |
| 16 weeks | *x.x (x.x), n* | *x.x (x.x), n* | *x.x (x.x to x.x)* | *x.xx* | *x.x (x.x), n* | *x.x (x.x), n* | *x.x (x.x to x.x)* | *x.xx* |
| 26 weeks | *x.x (x.x), n* | *x.x (x.x), n* | *x.x (x.x to x.x)* | *x.xx* | *x.x (x.x), n* | *x.x (x.x), n* | *x.x (x.x to x.x)* | *x.xx* |
| 52 weeks | *x.x (x.x), n* | *x.x (x.x), n* | *x.x (x.x to x.x)* | *x.xx* | *x.x (x.x), n* | *x.x (x.x), n* | *x.x (x.x to x.x)* | *x.xx* |
| **PROMIS Pain interference** | | | | | | | | |
| Baseline | *xx.x (xx.x), n* | *xx.x (xx.x), n* |  |  | *xx.x (xx.x), n* | *xx.x (xx.x), n* |  |  |
| 16 weeks | *xx.x (xx.x), n* | *xx.x (xx.x), n* | *xx.x (xx.x to xx.x)* | *x.xx* | *xx.x (xx.x), n* | *xx.x (xx.x), n* | *xx.x (xx.x to xx.x)* | *x.xx* |
| 26 weeks | *xx.x (xx.x), n* | *xx.x (xx.x), n* | *xx.x (xx.x to xx.x)* | *x.xx* | *xx.x (xx.x), n* | *xx.x (xx.x), n* | *xx.x (xx.x to xx.x)* | *x.xx* |
| 52 weeks | *xx.x (xx.x), n* | *xx.x (xx.x), n* | *xx.x (xx.x to xx.x)* | *x.xx* | *xx.x (xx.x), n* | *xx.x (xx.x), n* | *xx.x (xx.x to xx.x)* | *x.xx* |
| **Physical function** | | | | | | | | |
| Baseline | *xx.x (xx.x), n* | *xx.x (xx.x), n* |  |  | *xx.x (xx.x), n* | *xx.x (xx.x), n* |  |  |
| 16 weeks | *xx.x (xx.x), n* | *xx.x (xx.x), n* | *xx.x (xx.x to xx.x)* | *x.xx* | *xx.x (xx.x), n* | *xx.x (xx.x), n* | *xx.x (xx.x to xx.x)* | *x.xx* |
| 26 weeks | *xx.x (xx.x), n* | *xx.x (xx.x), n* | *xx.x (xx.x to xx.x)* | *x.xx* | *xx.x (xx.x), n* | *xx.x (xx.x), n* | *xx.x (xx.x to xx.x)* | *x.xx* |
| 52 weeks | *xx.x (xx.x), n* | *xx.x (xx.x), n* | *xx.x (xx.x to xx.x)* | *x.xx* | *xx.x (xx.x), n* | *xx.x (xx.x), n* | *xx.x (xx.x to xx.x)* | *x.xx* |
| **Fatigue** | | | | | | | | |
| Baseline | *xx.x (xx.x), n* | *xx.x (xx.x), n* |  |  | *xx.x (xx.x), n* | *xx.x (xx.x), n* |  |  |
| 16 weeks | *xx.x (xx.x), n* | *xx.x (xx.x), n* | *xx.x (xx.x to xx.x)* | *x.xx* | *xx.x (xx.x), n* | *xx.x (xx.x), n* | *xx.x (xx.x to xx.x)* | *x.xx* |
| 26 weeks | *xx.x (xx.x), n* | *xx.x (xx.x), n* | *xx.x (xx.x to xx.x)* | *x.xx* | *xx.x (xx.x), n* | *xx.x (xx.x), n* | *xx.x (xx.x to xx.x)* | *x.xx* |
| 52 weeks | *xx.x (xx.x), n* | *xx.x (xx.x), n* | *xx.x (xx.x to xx.x)* | *x.xx* | *xx.x (xx.x), n* | *xx.x (xx.x), n* | *xx.x (xx.x to xx.x)* | *x.xx* |
| **Self-efficacy to manage symptoms** | | | | | | | | |
| Baseline | *xx.x (xx.x), n* | *xx.x (xx.x), n* |  |  | *xx.x (xx.x), n* | *xx.x (xx.x), n* |  |  |
| 16 weeks | *xx.x (xx.x), n* | *xx.x (xx.x), n* | *xx.x (xx.x to xx.x)* | *x.xx* | *xx.x (xx.x), n* | *xx.x (xx.x), n* | *xx.x (xx.x to xx.x)* | *x.xx* |
| 26 weeks | *xx.x (xx.x), n* | *xx.x (xx.x), n* | *xx.x (xx.x to xx.x)* | *x.xx* | *xx.x (xx.x), n* | *xx.x (xx.x), n* | *xx.x (xx.x to xx.x)* | *x.xx* |
| 52 weeks | *xx.x (xx.x), n* | *xx.x (xx.x), n* | *xx.x (xx.x to xx.x)* | *x.xx* | *xx.x (xx.x), n* | *xx.x (xx.x), n* | *xx.x (xx.x to xx.x)* | *x.xx* |
| **Cognitive function** | | | | | | | | |
| Baseline | *xx.x (xx.x), n* | *xx.x (xx.x), n* |  |  | *xx.x (xx.x), n* | *xx.x (xx.x), n* |  |  |
| 16 weeks | *xx.x (xx.x), n* | *xx.x (xx.x), n* | *xx.x (xx.x to xx.x)* | *x.xx* | *xx.x (xx.x), n* | *xx.x (xx.x), n* | *xx.x (xx.x to xx.x)* | *x.xx* |
| 26 weeks | *xx.x (xx.x), n* | *xx.x (xx.x), n* | *xx.x (xx.x to xx.x)* | *x.xx* | *xx.x (xx.x), n* | *xx.x (xx.x), n* | *xx.x (xx.x to xx.x)* | *x.xx* |
| 52 weeks | *xx.x (xx.x), n* | *xx.x (xx.x), n* | *xx.x (xx.x to xx.x)* | *x.xx* | *xx.x (xx.x), n* | *xx.x (xx.x), n* | *xx.x (xx.x to xx.x)* | *x.xx* |
| **Depressive symptoms** | | | | | | | | |
| Baseline | *xx.x (xx.x), n* | *xx.x (xx.x), n* |  |  | *xx.x (xx.x), n* | *xx.x (xx.x), n* |  |  |
| 16 weeks | *xx.x (xx.x), n* | *xx.x (xx.x), n* | *xx.x (xx.x to xx.x)* | *x.xx* | *xx.x (xx.x), n* | *xx.x (xx.x), n* | *xx.x (xx.x to xx.x)* | *x.xx* |
| 26 weeks | *xx.x (xx.x), n* | *xx.x (xx.x), n* | *xx.x (xx.x to xx.x)* | *x.xx* | *xx.x (xx.x), n* | *xx.x (xx.x), n* | *xx.x (xx.x to xx.x)* | *x.xx* |
| 52 weeks | *xx.x (xx.x), n* | *xx.x (xx.x), n* | *xx.x (xx.x to xx.x)* | *x.xx* | *xx.x (xx.x), n* | *xx.x (xx.x), n* | *xx.x (xx.x to xx.x)* | *x.xx* |
| **Health-related quality of life** | | | | | | | | |
| Baseline | *xx.x (xx.x), n* | *xx.x (xx.x), n* |  |  | *xx.x (xx.x), n* | *xx.x (xx.x), n* |  |  |
| 16 weeks | *xx.x (xx.x), n* | *xx.x (xx.x), n* | *xx.x (xx.x to xx.x)* | *x.xx* | *xx.x (xx.x), n* | *xx.x (xx.x), n* | *xx.x (xx.x to xx.x)* | *x.xx* |
| 52 weeks | *xx.x (xx.x), n* | *xx.x (xx.x), n* | *xx.x (xx.x to xx.x)* | *x.xx* | *xx.x (xx.x), n* | *xx.x (xx.x), n* | *xx.x (xx.x to xx.x)* | *x.xx* |
| **Pain self-efficacy** | | | | | | | | |
| Baseline | *xx.x (xx.x), n* | *xx.x (xx.x), n* |  |  | *xx.x (xx.x), n* | *xx.x (xx.x), n* |  |  |
| 16 weeks | *x.x (x.x), n* | *x.x (x.x), n* | *x.x (x.x to x.x)* | *x.xx* | *x.x (x.x), n* | *x.x (x.x), n* | *x.x (x.x to x.x)* | *x.xx* |
| 26 weeks | *x.x (x.x), n* | *x.x (x.x), n* | *x.x (x.x to x.x)* | *x.xx* | *x.x (x.x), n* | *x.x (x.x), n* | *x.x (x.x to x.x)* | *x.xx* |
| 52 weeks | *x.x (x.x), n* | *x.x (x.x), n* | *x.x (x.x to x.x)* | *x.xx* | *x.x (x.x), n* | *x.x (x.x), n* | *x.x (x.x to x.x)* | *x.xx* |
| **Patient global impression of change** | | | | | | | | |
| 16 weeks | *x.x (x.x), n* | *x.x (x.x), n* | *x.x (x.x to x.x)* | *x.xx* | *x.x (x.x), n* | *x.x (x.x), n* | *x.x (x.x to x.x)* | *x.xx* |
| **CRPS severity** | | | | | | | | |
| Baseline | *xx.x (xx.x), n* | *xx.x (xx.x), n* |  |  | *xx.x (xx.x), n* | *xx.x (xx.x), n* |  |  |
| 16 weeks | *xx.x (xx.x), n* | *xx.x (xx.x), n* | *xx.x (xx.x to xx.x)* | *x.xx* | *xx.x (xx.x), n* | *xx.x (xx.x), n* | *xx.x (xx.x to xx.x)* | *x.xx* |

CRPS: complex regional pain syndrome.

**Supplementary table 3: adverse events for memantine and placebo**

|  | **During treatment** | | | | **Post-treatment** | | | |
| --- | --- | --- | --- | --- | --- | --- | --- | --- |
|  | **Memantine** | **Placebo** | **Risk ratio** | **P value** | **Memantine** | **Placebo** | **Risk ratio** | **P value** |
| Participants with ≥1 AEs | *n/N (%)* | *n/N (%)* | *x.xx (x.xx to x.xx)* | *x.xx* | *n/N (%)* | *n/N (%)* | *x.xx (x.xx to x.xx)* | *x.xx* |
| Participants with ≥1 SAEs | *n/N (%)* | *n/N (%)* | *x.xx (x.xx to x.xx)* | *x.xx* | *n/N (%)* | *n/N (%)* | *x.xx (x.xx to x.xx)* | *x.xx* |
| Total number of SAEs | *n/N (%)* | *n/N (%)* | *x.xx (x.xx to x.xx)* | *x.xx* | *n/N (%)* | *n/N (%)* | *x.xx (x.xx to x.xx)* | *x.xx* |
| Related SAEs | *n/N (%)* | *n/N (%)* | *x.xx (x.xx to x.xx)* | *x.xx* | *n/N (%)* | *n/N (%)* | *x.xx (x.xx to x.xx)* | *x.xx* |
| Unrelated SAEs | *n/N (%)* | *n/N (%)* | *x.xx (x.xx to x.xx)* | *x.xx* | *n/N (%)* | *n/N (%)* | *x.xx (x.xx to x.xx)* | *x.xx* |
| Tiredness | *n/N (%)* | *n/N (%)* | *x.xx (x.xx to x.xx)* | *x.xx* | *n/N (%)* | *n/N (%)* | *x.xx (x.xx to x.xx)* | *x.xx* |
| Sleeplessness | *n/N (%)* | *n/N (%)* | *x.xx (x.xx to x.xx)* | *x.xx* | *n/N (%)* | *n/N (%)* | *x.xx (x.xx to x.xx)* | *x.xx* |
| Dizziness | *n/N (%)* | *n/N (%)* | *x.xx (x.xx to x.xx)* | *x.xx* | *n/N (%)* | *n/N (%)* | *x.xx (x.xx to x.xx)* | *x.xx* |
| Falls | *n/N (%)* | *n/N (%)* | *x.xx (x.xx to x.xx)* | *x.xx* | *n/N (%)* | *n/N (%)* | *x.xx (x.xx to x.xx)* | *x.xx* |
| Diarrhoea | *n/N (%)* | *n/N (%)* | *x.xx (x.xx to x.xx)* | *x.xx* | *n/N (%)* | *n/N (%)* | *x.xx (x.xx to x.xx)* | *x.xx* |
| Vomiting | *n/N (%)* | *n/N (%)* | *x.xx (x.xx to x.xx)* | *x.xx* | *n/N (%)* | *n/N (%)* | *x.xx (x.xx to x.xx)* | *x.xx* |
| Nausea | *n/N (%)* | *n/N (%)* | *x.xx (x.xx to x.xx)* | *x.xx* | *n/N (%)* | *n/N (%)* | *x.xx (x.xx to x.xx)* | *x.xx* |
| Anorexia | *n/N (%)* | *n/N (%)* | *x.xx (x.xx to x.xx)* | *x.xx* | *n/N (%)* | *n/N (%)* | *x.xx (x.xx to x.xx)* | *x.xx* |
| Anxiety | *n/N (%)* | *n/N (%)* | *x.xx (x.xx to x.xx)* | *x.xx* | *n/N (%)* | *n/N (%)* | *x.xx (x.xx to x.xx)* | *x.xx* |
| Headache | *n/N (%)* | *n/N (%)* | *x.xx (x.xx to x.xx)* | *x.xx* | *n/N (%)* | *n/N (%)* | *x.xx (x.xx to x.xx)* | *x.xx* |
| Confusion | *n/N (%)* | *n/N (%)* | *x.xx (x.xx to x.xx)* | *x.xx* | *n/N (%)* | *n/N (%)* | *x.xx (x.xx to x.xx)* | *x.xx* |
| Hallucinations | *n/N (%)* | *n/N (%)* | *x.xx (x.xx to x.xx)* | *x.xx* | *n/N (%)* | *n/N (%)* | *x.xx (x.xx to x.xx)* | *x.xx* |
| Delusions | *n/N (%)* | *n/N (%)* | *x.xx (x.xx to x.xx)* | *x.xx* | *n/N (%)* | *n/N (%)* | *x.xx (x.xx to x.xx)* | *x.xx* |

AE: adverse event; SAE: serious adverse event

**Supplementary table 4: adverse events for graded motor imagery and no graded motor imagery**

|  | **During treatment** | | | | **Post-treatment** | | | |
| --- | --- | --- | --- | --- | --- | --- | --- | --- |
|  | **GMI** | **No GMI** | **Risk ratio** | **P value** | **GMI** | **No GMI** | **Risk ratio** | **P value** |
| Participants with ≥1 AEs | *n/N (%)* | *n/N (%)* | *x.xx (x.xx to x.xx)* | *x.xx* | *n/N (%)* | *n/N (%)* | *x.xx (x.xx to x.xx)* | *x.xx* |
| Participants with ≥1 SAEs | *n/N (%)* | *n/N (%)* | *x.xx (x.xx to x.xx)* | *x.xx* | *n/N (%)* | *n/N (%)* | *x.xx (x.xx to x.xx)* | *x.xx* |
| Total number of SAEs | *n/N (%)* | *n/N (%)* | *x.xx (x.xx to x.xx)* | *x.xx* | *n/N (%)* | *n/N (%)* | *x.xx (x.xx to x.xx)* | *x.xx* |
| Related SAEs | *n/N (%)* | *n/N (%)* | *x.xx (x.xx to x.xx)* | *x.xx* | *n/N (%)* | *n/N (%)* | *x.xx (x.xx to x.xx)* | *x.xx* |
| Unrelated SAEs | *n/N (%)* | *n/N (%)* | *x.xx (x.xx to x.xx)* | *x.xx* | *n/N (%)* | *n/N (%)* | *x.xx (x.xx to x.xx)* | *x.xx* |

AE: adverse event; GMI: graded motor imagery; SAE: serious adverse event

**Supplementary table 5: medicine use**

|  | **Memantine** | **Placebo** | **Risk ratio** | **p value** | **Graded motor imagery** | **No graded motor imagery** | **Risk ratio** | **p value** |
| --- | --- | --- | --- | --- | --- | --- | --- | --- |
| **Prescription medicines** | | | | | | | | |
| Strong opioids |  |  |  |  |  |  |  |  |
| 16 weeks | *n/N (%)* | *n/N (%)* | *x.xx (x.xx to x.xx)* | *x.xx* | *n/N (%)* | *n/N (%)* | *x.xx (x.xx to x.xx)* | *x.xx* |
| 26 weeks | *n/N (%)* | *n/N (%)* | *x.xx (x.xx to x.xx)* | *x.xx* | *n/N (%)* | *n/N (%)* | *x.xx (x.xx to x.xx)* | *x.xx* |
| 52 weeks | *n/N (%)* | *n/N (%)* | *x.xx (x.xx to x.xx)* | *x.xx* | *n/N (%)* | *n/N (%)* | *x.xx (x.xx to x.xx)* | *x.xx* |
| Weak opioids | | | | | | | | |
| 16 weeks | *n/N (%)* | *n/N (%)* | *x.xx (x.xx to x.xx)* | *x.xx* | *n/N (%)* | *n/N (%)* | *x.xx (x.xx to x.xx)* | *x.xx* |
| 26 weeks | *n/N (%)* | *n/N (%)* | *x.xx (x.xx to x.xx)* | *x.xx* | *n/N (%)* | *n/N (%)* | *x.xx (x.xx to x.xx)* | *x.xx* |
| 52 weeks | *n/N (%)* | *n/N (%)* | *x.xx (x.xx to x.xx)* | *x.xx* | *n/N (%)* | *n/N (%)* | *x.xx (x.xx to x.xx)* | *x.xx* |
| Anticonvulsants | | | | | | | | |
| 16 weeks | *n/N (%)* | *n/N (%)* | *x.xx (x.xx to x.xx)* | *x.xx* | *n/N (%)* | *n/N (%)* | *x.xx (x.xx to x.xx)* | *x.xx* |
| 26 weeks | *n/N (%)* | *n/N (%)* | *x.xx (x.xx to x.xx)* | *x.xx* | *n/N (%)* | *n/N (%)* | *x.xx (x.xx to x.xx)* | *x.xx* |
| 52 weeks | *n/N (%)* | *n/N (%)* | *x.xx (x.xx to x.xx)* | *x.xx* | *n/N (%)* | *n/N (%)* | *x.xx (x.xx to x.xx)* | *x.xx* |
| Antidepressants | | | | | | | | |
| 16 weeks | *n/N (%)* | *n/N (%)* | *x.xx (x.xx to x.xx)* | *x.xx* | *n/N (%)* | *n/N (%)* | *x.xx (x.xx to x.xx)* | *x.xx* |
| 26 weeks | *n/N (%)* | *n/N (%)* | *x.xx (x.xx to x.xx)* | *x.xx* | *n/N (%)* | *n/N (%)* | *x.xx (x.xx to x.xx)* | *x.xx* |
| 52 weeks | *n/N (%)* | *n/N (%)* | *x.xx (x.xx to x.xx)* | *x.xx* | *n/N (%)* | *n/N (%)* | *x.xx (x.xx to x.xx)* | *x.xx* |
| Corticosteroids | | | | | | | | |
| 16 weeks | *n/N (%)* | *n/N (%)* | *x.xx (x.xx to x.xx)* | *x.xx* | *n/N (%)* | *n/N (%)* | *x.xx (x.xx to x.xx)* | *x.xx* |
| 26 weeks | *n/N (%)* | *n/N (%)* | *x.xx (x.xx to x.xx)* | *x.xx* | *n/N (%)* | *n/N (%)* | *x.xx (x.xx to x.xx)* | *x.xx* |
| 52 weeks | *n/N (%)* | *n/N (%)* | *x.xx (x.xx to x.xx)* | *x.xx* | *n/N (%)* | *n/N (%)* | *x.xx (x.xx to x.xx)* | *x.xx* |
| NSAIDs | | | | | | | | |
| 16 weeks | *n/N (%)* | *n/N (%)* | *x.xx (x.xx to x.xx)* | *x.xx* | *n/N (%)* | *n/N (%)* | *x.xx (x.xx to x.xx)* | *x.xx* |
| 26 weeks | *n/N (%)* | *n/N (%)* | *x.xx (x.xx to x.xx)* | *x.xx* | *n/N (%)* | *n/N (%)* | *x.xx (x.xx to x.xx)* | *x.xx* |
| 52 weeks | *n/N (%)* | *n/N (%)* | *x.xx (x.xx to x.xx)* | *x.xx* | *n/N (%)* | *n/N (%)* | *x.xx (x.xx to x.xx)* | *x.xx* |
| **Over the counter medicines** | | | | | | | | |
| Paracetamol |  |  |  |  |  |  |  |  |
| 16 weeks | *n/N (%)* | *n/N (%)* | *x.xx (x.xx to x.xx)* | *x.xx* | *n/N (%)* | *n/N (%)* | *x.xx (x.xx to x.xx)* | *x.xx* |
| 26 weeks | *n/N (%)* | *n/N (%)* | *x.xx (x.xx to x.xx)* | *x.xx* | *n/N (%)* | *n/N (%)* | *x.xx (x.xx to x.xx)* | *x.xx* |
| 52 weeks | *n/N (%)* | *n/N (%)* | *x.xx (x.xx to x.xx)* | *x.xx* | *n/N (%)* | *n/N (%)* | *x.xx (x.xx to x.xx)* | *x.xx* |
| NSAIDs |  |  |  |  |  |  |  |  |
| 16 weeks | *n/N (%)* | *n/N (%)* | *x.xx (x.xx to x.xx)* | *x.xx* | *n/N (%)* | *n/N (%)* | *x.xx (x.xx to x.xx)* | *x.xx* |
| 26 weeks | *n/N (%)* | *n/N (%)* | *x.xx (x.xx to x.xx)* | *x.xx* | *n/N (%)* | *n/N (%)* | *x.xx (x.xx to x.xx)* | *x.xx* |
| 52 weeks | *n/N (%)* | *n/N (%)* | *x.xx (x.xx to x.xx)* | *x.xx* | *n/N (%)* | *n/N (%)* | *x.xx (x.xx to x.xx)* | *x.xx* |
| Topicals |  |  |  |  |  |  |  |  |
| 16 weeks | *n/N (%)* | *n/N (%)* | *x.xx (x.xx to x.xx)* | *x.xx* | *n/N (%)* | *n/N (%)* | *x.xx (x.xx to x.xx)* | *x.xx* |
| 26 weeks | *n/N (%)* | *n/N (%)* | *x.xx (x.xx to x.xx)* | *x.xx* | *n/N (%)* | *n/N (%)* | *x.xx (x.xx to x.xx)* | *x.xx* |
| 52 weeks | *n/N (%)* | *n/N (%)* | *x.xx (x.xx to x.xx)* | *x.xx* | *n/N (%)* | *n/N (%)* | *x.xx (x.xx to x.xx)* | *x.xx* |
|  |  |  |  |  |  |  |  |  |

**Supplementary table 6: multi-arm sensitivity analysis and interaction effects for primary and secondary outcomes**

|  | **Memantine & graded motor imagery versus placebo only** | | **Memantine only versus placebo only** | | **Placebo and graded motor imagery versus placebo only** | | **Interaction (memantine & graded motor imagery)** | |
| --- | --- | --- | --- | --- | --- | --- | --- | --- |
|  | **Mean difference** | **p value** | **Mean difference** | **p value** | **Mean difference** | **p value** | **Interaction effect** | **p value** |
| **Pain intensity** | | | | | | | | |
| 16 weeks | *x.x (x.x to x.x)* | *x.xx* | *x.x (x.x to x.x)* | *x.xx* | *x.x (x.x to x.x)* | *x.xx* | *x.x (x.x to x.x)* | *x.xx* |
| 26 weeks | *x.x (x.x to x.x)* | *x.xx* | *x.x (x.x to x.x)* | *x.xx* | *x.x (x.x to x.x)* | *x.xx* | *x.x (x.x to x.x)* | *x.xx* |
| 52 weeks | *x.x (x.x to x.x)* | *x.xx* | *x.x (x.x to x.x)* | *x.xx* | *x.x (x.x to x.x)* | *x.xx* | *x.x (x.x to x.x)* | *x.xx* |
| **Pain interference** |  |  |  |  |  |  |  |  |
| 16 weeks | *xx.x (xx.x to xx.x)* | *x.xx* | *xx.x (xx.x to xx.x)* | *x.xx* | *xx.x (xx.x to xx.x)* | *x.xx* | *xx.x (xx.x to xx.x)* | *x.xx* |
| 26 weeks | *xx.x (xx.x to xx.x)* | *x.xx* | *xx.x (xx.x to xx.x)* | *x.xx* | *xx.x (xx.x to xx.x)* | *x.xx* | *xx.x (xx.x to xx.x)* | *x.xx* |
| 52 weeks | *xx.x (xx.x to xx.x)* | *x.xx* | *xx.x (xx.x to xx.x)* | *x.xx* | *xx.x (xx.x to xx.x)* | *x.xx* | *xx.x (xx.x to xx.x)* | *x.xx* |
| **Physical function** | | | | | | | | |
| 16 weeks | *xx.x (xx.x to xx.x)* | *x.xx* | *xx.x (xx.x to xx.x)* | *x.xx* | *xx.x (xx.x to xx.x)* | *x.xx* | *xx.x (xx.x to xx.x)* | *x.xx* |
| 26 weeks | *xx.x (xx.x to xx.x)* | *x.xx* | *xx.x (xx.x to xx.x)* | *x.xx* | *xx.x (xx.x to xx.x)* | *x.xx* | *xx.x (xx.x to xx.x)* | *x.xx* |
| 52 weeks | *xx.x (xx.x to xx.x)* | *x.xx* | *xx.x (xx.x to xx.x)* | *x.xx* | *xx.x (xx.x to xx.x)* | *x.xx* | *xx.x (xx.x to xx.x)* | *x.xx* |
| **Fatigue** | | | | | | | | |
| 16 weeks | *xx.x (xx.x to xx.x)* | *x.xx* | *xx.x (xx.x to xx.x)* | *x.xx* | *xx.x (xx.x to xx.x)* | *x.xx* | *xx.x (xx.x to xx.x)* | *x.xx* |
| 26 weeks | *xx.x (xx.x to xx.x)* | *x.xx* | *xx.x (xx.x to xx.x)* | *x.xx* | *xx.x (xx.x to xx.x)* | *x.xx* | *xx.x (xx.x to xx.x)* | *x.xx* |
| 52 weeks | *xx.x (xx.x to xx.x)* | *x.xx* | *xx.x (xx.x to xx.x)* | *x.xx* | *xx.x (xx.x to xx.x)* | *x.xx* | *xx.x (xx.x to xx.x)* | *x.xx* |
| **Self-efficacy to manage symptoms** | | | | | | | | |
| 16 weeks | *xx.x (xx.x to xx.x)* | *x.xx* | *xx.x (xx.x to xx.x)* | *x.xx* | *xx.x (xx.x to xx.x)* | *x.xx* | *xx.x (xx.x to xx.x)* | *x.xx* |
| 26 weeks | *xx.x (xx.x to xx.x)* | *x.xx* | *xx.x (xx.x to xx.x)* | *x.xx* | *xx.x (xx.x to xx.x)* | *x.xx* | *xx.x (xx.x to xx.x)* | *x.xx* |
| 52 weeks | *xx.x (xx.x to xx.x)* | *x.xx* | *xx.x (xx.x to xx.x)* | *x.xx* | *xx.x (xx.x to xx.x)* | *x.xx* | *xx.x (xx.x to xx.x)* | *x.xx* |
| **Cognitive function** | | | | | | | | |
| Baseline | *xx.x (xx.x to xx.x)* | *x.xx* | *xx.x (xx.x to xx.x)* | *x.xx* | *xx.x (xx.x to xx.x)* | *x.xx* | *xx.x (xx.x to xx.x)* | *x.xx* |
| 26 weeks | *xx.x (xx.x to xx.x)* | *x.xx* | *xx.x (xx.x to xx.x)* | *x.xx* | *xx.x (xx.x to xx.x)* | *x.xx* | *xx.x (xx.x to xx.x)* | *x.xx* |
| 52 weeks | *xx.x (xx.x to xx.x)* | *x.xx* | *xx.x (xx.x to xx.x)* | *x.xx* | *xx.x (xx.x to xx.x)* | *x.xx* | *xx.x (xx.x to xx.x)* | *x.xx* |
| **Depressive symptoms** | | | | | | | | |
| 16 weeks | *xx.x (xx.x to xx.x)* | *x.xx* | *xx.x (xx.x to xx.x)* | *x.xx* | *xx.x (xx.x to xx.x)* | *x.xx* | *xx.x (xx.x to xx.x)* | *x.xx* |
| 26 weeks | *xx.x (xx.x to xx.x)* | *x.xx* | *xx.x (xx.x to xx.x)* | *x.xx* | *xx.x (xx.x to xx.x)* | *x.xx* | *xx.x (xx.x to xx.x)* | *x.xx* |
| 52 weeks | *xx.x (xx.x to xx.x)* | *x.xx* | *xx.x (xx.x to xx.x)* | *x.xx* | *xx.x (xx.x to xx.x)* | *x.xx* | *xx.x (xx.x to xx.x)* | *x.xx* |
| **Health-related quality of life** | | | | | | | | |
| 16 weeks | *xx.x (xx.x to xx.x)* | *x.xx* | *xx.x (xx.x to xx.x)* | *x.xx* | *xx.x (xx.x to xx.x)* | *x.xx* | *xx.x (xx.x to xx.x)* | *x.xx* |
| 52 weeks | *xx.x (xx.x to xx.x)* | *x.xx* | *xx.x (xx.x to xx.x)* | *x.xx* | *xx.x (xx.x to xx.x)* | *x.xx* | *xx.x (xx.x to xx.x)* | *x.xx* |
| **Pain self-efficacy** | | | | | | | | |
| 16 weeks | *x.x (x.x to x.x)* | *x.xx* | *x.x (x.x to x.x)* | *x.xx* | *x.x (x.x to x.x)* | *x.xx* | *x.x (x.x to x.x)* | *x.xx* |
| 26 weeks | *x.x (x.x to x.x)* | *x.xx* | *x.x (x.x to x.x)* | *x.xx* | *x.x (x.x to x.x)* | *x.xx* | *x.x (x.x to x.x)* | *x.xx* |
| 52 weeks | *x.x (x.x to x.x)* | *x.xx* | *x.x (x.x to x.x)* | *x.xx* | *x.x (x.x to x.x)* | *x.xx* | *x.x (x.x to x.x)* | *x.xx* |
| **Patient global impression of change** | | | | | | | | |
| 16 weeks | *x.x (x.x), n* | *x.x (x.x), n* | *x.x (x.x to x.x)* | *x.xx* | *x.x (x.x to x.x)* | *x.xx* | *x.x (x.x to x.x)* | *x.xx* |
| **CRPS severity** |  |  |  |  |  |  |  |  |
| 16 weeks | *xx.x (xx.x to xx.x)* | *x.xx* | *xx.x (xx.x to xx.x)* | *x.xx* | *xx.x (xx.x to xx.x)* | *x.xx* | *xx.x (xx.x to xx.x)* | *x.xx* |

**Appendix 1: Intervention adherence**

A summary of the interventions allocated and received will be reported for each of the four intervention groups (Supplementary table 7). Adherence to the memantine and placebo groups will be summarised using the following variables:

- Number of treatment discontinuations
- Maintenance dose
- Percentage of prescribed dose taken
- Percentage of returned medicines

Adherence to the graded motor imagery intervention will be summarised using the following variables:

- Number of treatment discontinuations
- Number of sessions attended
- Time spent doing home activities

**Supplementary table 7: intervention adherence**

|  | **Comparison 1** | | **Comparison 2** | |
| --- | --- | --- | --- | --- |
|  | **Memantine (n=)** | **Placebo (n=)** | **Graded motor imagery (n=)** | **No graded motor imagery (n =)** |
| Maintenance dose | | |  |  |
| 5mg/day | *n/N (%)* | *n/N (%)* | *-* | *-* |
| 10mg/day | *n/N (%)* | *n/N (%)* | *-* | *-* |
| 15mg/day | *n/N (%)* | *n/N (%)* | *-* | *-* |
| 20mg/day | *n/N (%)* | *n/N (%)* | *-* | *-* |
| 25mg/day | *n/N (%)* | *n/N (%)* | *-* | *-* |
| 30mg/day | *n/N (%)* | *n/N (%)* | *-* | *-* |
| 35mg/day | *n/N (%)* | *n/N (%)* | *-* | *-* |
| 40mg/day | *n/N (%)* | *n/N (%)* | *-* | *-* |
| Returned medicines | *n/N (%)* | *n/N (%)* | *-* | *-* |
| <20% | *n/N (%)* | *n/N (%)* |  |  |
| 20 to <40% | *n/N (%)* | *n/N (%)* |  |  |
| 40% to <60% | *n/N (%)* | *n/N (%)* |  |  |
| 60% to <80% | *n/N (%)* | *n/N (%)* |  |  |
| ≥80% | *n/N (%)* | *n/N (%)* |  |  |
| Sessions completed | *-* | *-* | *x.x (SD), n* | *x.x (SD), n* |
| 1 | *-* | *-* | *n/N (%)* | *n/N (%)* |
| 2 | *-* | *-* | *n/N (%)* | *n/N (%)* |
| 3 | *-* | *-* | *n/N (%)* | *n/N (%)* |
| 4 | *-* | *-* | *n/N (%)* | *n/N (%)* |
| 5 | *-* | *-* | *n/N (%)* | *n/N (%)* |
| 6 | *-* | *-* | *n/N (%)* | *n/N (%)* |
| 7 | *-* | *-* | *n/N (%)* | *n/N (%)* |
| Time on home activities | *-* | *-* | *xx.x (SD), n* | *xx.x (SD), n* |

**Appendix 2: Protocol deviations**

Protocol deviations are defined as any breach of the study protocol. For the MEMOIR trial, these relate to eligibility criteria, use of prohibited concomitant treatments, and study conduct. Deviations will be summarised for each treatment group (Supplementary table 8); participants that are included in the ITT analysis data set will be used as the denominator to calculate the percentages.

**Supplementary table 8: Protocol deviations**

|  | **Comparison 1** |  | **Comparison 2** |  |
| --- | --- | --- | --- | --- |
|  | **Placebo (n=)** | **Graded motor imagery (n=)** | **Placebo (n=)** | **Graded motor imagery (n=)** |
| Did not fulfil eligibility criteria | | | | |
| Reason 1 | *n/N (%)* | *n/N (%)* | *n/N (%)* | *n/N (%)* |
| Reason 2 | *n/N (%)* | *n/N (%)* | *n/N (%)* | *n/N (%)* |
| Use of prohibited concomitant treatments | | |  |  |
| Treatment 1 | *n/N (%)* | *n/N (%)* | *n/N (%)* | *n/N (%)* |
| Treatment 2 | *n/N (%)* | *n/N (%)* | *n/N (%)* | *n/N (%)* |
| Study conduct | | | | |
| Reason 1 | *n/N (%)* | *n/N (%)* | *n/N (%)* | *n/N (%)* |
| Reason 2 | *n/N (%)* | *n/N (%)* | *n/N (%)* | *n/N (%)* |
